# The Health Benefits of Nutrients and Bioactive Compounds in Functional Foods and Beverages

**DOI:** 10.3390/nu17233679

**Published:** 2025-11-25

**Authors:** Simona Ioana Vicas

**Affiliations:** 1 Department of Food Engineering, University of Oradea, 26 Gen. Magheru St., 410048 Oradea, Romania; svicas@uoradea.ro; 2 Biomedical Sciences Doctoral School, University of Oradea, 1 University St., 410087 Oradea, Romania

## 1. Introduction

Functional foods and beverages enriched with nutrients and bioactive compounds have become a cornerstone in promoting health and preventing chronic diseases, marking a paradigm shift from basic nutrition toward diets with proven physiological benefits. In recent years, research has advanced from simply identifying bioactive molecules to understanding their mechanisms of action, bioavailability, and synergistic interactions within complex food matrices.

Despite these research advancements, there remain gaps in translating promising research findings into real-world benefits. These include unstable compounds, low bioavailability, variability in diverse natural sources, and a lack of uniform regulatory frameworks. Often, clinical evidence is insufficient to support health claims, and the absence of defined criteria across countries complicates research and product development.

Some of these gaps have started to close thanks to new technologies. Microencapsulation, nanoemulsions, and nanoparticles made of proteins or polymers have shown promise in protecting unstable substances and enhancing their intestinal uptake. These delivery systems can make polyphenols, vitamins, and probiotics more stable while they are being processed and digested, which could potentially increase their functional efficacy. However, these potential benefits at the consumer level require rigorous clinical validation and safety evaluation.

Consumer perceptions and regulatory clarity are just as essential. Functional foods must not only provide validated benefits but also meet requirements regarding naturalness, flavor, and labeling integrity. Consistent scientific evidence, clear communication, and harmonious regulatory procedures are essential for building trust in functional foods and beverages.

The Special Issue, “The Health Benefits of Nutrients and Bioactive Compounds in Functional Foods and Beverages”, was designed to bridge existing knowledge gaps by compiling new experimental and review studies. The six published articles [1,2,3,4,5,6], comprising four original research articles [2,4,5,6] and two reviews [1,3], underscore advancements in bioactive compounds, formulation strategies, and health-promoting uses. This Editorial succinctly summarizes their contributions, highlighting how they collectively strengthen the scientific foundations of this fast-advancing field and outline opportunities for future research.

## 2. An Overview of Published Articles

This Special Issue includes six contributions that investigate food-derived nutrients or fortified matrices with potential health benefits including mechanistic research, formulation science, and quality and safety assessments. They jointly focus on three interrelated themes: (i) gut barrier integrity, inflammation, and microbiota–metabolite interactions; (ii) delivery science for unstable or bio-limited bioactives; and (iii) content quality and regulatory readiness in consumer products.

### 2.1. Intestinal Health, Hypoxic Adaptation, and Inflammatory Control

Two original research papers [2,6] and one review [1] focus on the intestinal epithelium and host–microbe interactions.

Extracellular vesicles (CM-EVs) generated from camel milk are presented as a unique, nanoscale functional component that alleviates colonic damage during hypobaric hypoxia more efficiently than total camel milk [6]. CM-EVs restored mucosal barrier integrity, reestablished gut microbiota by enhancing beneficial (*Lactobacillus*, *Bifidobacterium*) and reducing harmful (*Enterobacteriaceae*) bacteria, and influenced bile-acid and amino-acid metabolism via FXR/NF-κB signaling, highlighting microbiota–metabolite interactions as a therapeutic pathway and suggesting a precision-nutrition delivery mechanism unique to food matrices. This based efficacy in conditions of environmental stress [6].

Purple potato extract (PP extract), a natural product rich in polyphenols, inhibits hypoxia-induced metabolic reprogramming in Caco-2 cells by suppressing HIF-1α and its transcriptional activator XBP1s, decreasing classical HIF-1 targets (GLUT1, LDHA, HK1, PDK1), and redirecting metabolism toward oxidative phosphorylation [2]. Functionally, PP extract inhibited proliferation and migration while diminishing 5-fluorouracil resistance in hypoxic conditions [2]. This finding highlights the distinction between the general abundance of polyphenols in natural products and the identification of specific, targetable molecular pathways through which selected compounds, such as those in PP extract, modulate colorectal cancer metabolism.

Chicken egg powder and bovine colostrum (BC), both alone and together, are examined in a review as potential GRAS nutraceuticals having antibacterial, immunomodulatory, and mucosal healing properties [1]. A significant conceptual development is the synergistic effect of an egg-to-BC ratio (approximately 40:60) that outperforms individual components in preclinical models of nonsteroidal anti-inflammatory drug (NSAID)-induced small-intestinal injury and (dextran sulfate sodium) DSS colitis [1]. Additionally, the review highlights emerging indications (e.g., GLP-1–associated gastrointestinal symptoms) and underscores the necessity for rigorous clinical trials [1]. This review reinterprets two well-known foods as a rational additional therapy and elucidates the areas where preclinical research requires human validation.

### 2.2. Delivery Science and Matrix Effects for Bioactives and Probiotics

Two contributions [3,4] examine the role of matrices and carriers in influencing the maintenance of bioactives during processing and gastrointestinal transit to provide benefits.

During simulated oral–gastric–intestinal digestion, dairy matrices provided better protection for *Lacticaseibacillus casei* and *Lactobacillus johnsonii* than oat drinks enriched with protein isolates [4]. Oat beverages, despite their fiber and β-glucan content, were less protective overall for these beneficial gut bacteria. In several formulations, the addition of plant isolates did not enhance the survivability of these probiotic bacteria throughout digestion. The research identifies matrix- and protein-specific limitations influencing probiotic administration during the shift to plant-based formats and establishes definitive formulation objectives for forthcoming, consumer-relevant products [4].

The CLA bone-health review integrates preclinical and clinical research on conjugated linoleic acid as an anti-inflammatory modulator of bone remodeling [3]. It also points out low solubility/absorption and oxidative instability as significant barriers to bioavailability. It lists nanoparticle-based methods (such as liposomes, NLCs, and cyclodextrins) that can improve delivery and suggests a multimodal approach, such as CLA plus electrical stimulation, to boost osteogenesis [3]. This review article addresses the ongoing gap between robust animal research and individual human results by establishing delivery and trial-design levers.

### 2.3. Quality, Safety, and Compliance with Regulations in Fortified Beverages

A market-wide study of vitamin-fortified non-alcoholic beverages (VFNABs) available on the Slovenian market revealed discrepancies between the labeled and the actual vitamin contents of these products [5]. About 48% of the vitamin levels that were analyzed examined were outside the EU’s acceptable range (65–150% of the label claim), with the lowest levels being 0% and the highest levels being 907% of the reported values [5]. The assessment revealed that these products generally meet the daily recommended intakes for vitamins, and in some cases, even exceed them substantially (up to 616% of the Nutrient Reference Values). This phenomenon is particularly concerning for children, who frequently consume large quantities of vitamin-fortified soft and energy drinks [5]. The study emphasizes the necessity for enhanced quality control, formulation prioritizing stability, and risk-appropriate labeling, directly addressing the regulatory-science divide in functional beverages.

## 3. Addressing Gaps in Knowledge in Functional Food Research

This Special Issue’s articles address various long-standing gaps in the field of functional foods, including those related to bioavailability, molecular mechanisms of action, formulation design, and product quality and safety.

A primary limitation in functional food research has been the inadequate stability and limited bioaccessibility of bioactive substances, which reduces their physiological effectiveness. Camel milk-derived extracellular vesicles are an example of an innovative delivery technology that can help address this limitation. These vesicles are natural nanocarriers that can protect and transport intact biomolecules over intestinal barriers [6]. The review on conjugated linoleic acid outlines the benefits of nanoparticle-based encapsulation (liposomes, nanostructured lipid carriers, and cyclodextrins) in enhancing solubility and targeted release [3]. Additionally, the combined application of egg powder and bovine colostrum represents a biological approach to protecting growth factors and immunoglobulins from enzymatic degradation during digestion [1].

Another persistent limitation in functional food research is the inadequate understanding of the molecular processes that underlie observed effects on health. The studies presented in this Special Issue significantly enhance mechanistic understanding by associating dietary bioactives with specific signaling pathways: the purple potato extract targets HIF-1α and XBP1s to alleviate hypoxia-induced metabolic reprogramming in colorectal cancer cells [2]; CM-EVs restore gut integrity through FXR/NF-κB modulation and microbiota–metabolite interaction [6]; and CLA is demonstrated to affect bone remodeling via anti-inflammatory cytokine suppression and Wnt/β-catenin signaling [3].

The Special Issue articles address the necessity for plant-based matrices and for making functional products work in present-day dietary and medical fields from both a formulation and a consumer perspective. The comparison of probiotic survival in milk versus oat-based beverages illustrates both the potential and current limitations of non-dairy carriers [4]. Meanwhile, the egg–colostrum combination presents a novel nutraceutical strategy for alleviating gastrointestinal adverse effects associated with GLP-1-based therapies, which is an emerging health issue [1].

Additionally, the article on non-alcoholic fortified beverages reveals a major gap in safety and regulation. It shows that almost half of the items tested had too much vitamin content for the EU’s tolerance levels, which could be dangerous for children, who often take supplemental vitamins. This article emphasizes the necessity for stringent analytical oversight, adherence to labeling regulations, and consumer protection initiatives [5].

Taken together, these Special Issue contributions shift the field from descriptive and compositional studies to a more integrated, mechanistic, and regulatory-aware approach to functional food development, one that combines bioactive delivery, molecular validation, and product reliability as the foundations of next-generation nutrition science.

## 4. Conclusions

The six articles in this Special Issue, which look at nutraceuticals, functional foods, and food safety, show that there is a definite shift toward mechanism-driven precision nutrition and the need for standardization and regulation. The articles encompass a range of subjects, including extracellular vesicles, probiotics, and fortified beverages, yet they converge on three principal conclusions.

First, the therapeutic potential of functional foods is dependent on their molecular mechanisms and delivery systems. The effectiveness of bioactive chemicals, including CLA, polyphenols, and growth factors, relies not alone on their existence but also on their stability, bioaccessibility, and precise delivery. Camel milk-derived extracellular vesicles and nanoparticle-based formulations of conjugated linoleic acid are two examples of innovative nanocarriers that assist digestive degradation and improve bioavailability. Likewise, synergistic formulations, such as egg powder in combination with bovine colostrum, provide biological preservation of sensitive bioactives and facilitate functional maintenance of the intestinal barrier.

Second, product quality and safety remain significant challenges. The investigation of fortified non-alcoholic beverages indicated considerable discrepancies between the reported vitamin levels and actual contents, alongside potential overexposure concerns, especially for children. This highlights the necessity for enhanced monitoring by regulators and accurate labeling.

Third, plant-based matrices for probiotic consumption require additional improvement. Despite increasing demand from consumers, dairy matrices continue to outperform non-dairy carriers in terms of probiotic viability throughout digestion, presenting an opportunity for future innovation.

In summary, this Special Issue highlights a paradigm shift in functional food science, from descriptive bioactive identification to an integrated model that connects molecular insight, delivery innovation, and regulatory rigor, all of which are critical pillars for advancing credible and effective functional nutrition.

## 5. Future Research Directions

Future research on functional foods and bioactive compounds will have to concentrate on converting mechanistic insights into clinically demonstrated outcomes. Priority directions encompass the standardization of bioactive compound characterization, the integration of multi-omics and systems biology to elucidate diet–microbiota–host interactions, and the advancement of validated delivery systems (nano- and microencapsulation) that exhibit enhanced bioefficacy and safety in humans. Long-term, real-world clinical trials aimed at dose–response interactions, population variability, and sustainability are just as critical. Finally, clear communication with consumers and standardized regulatory frameworks will be key to linking scientific progress with confidence among consumers. This will ensure that functional foods perform up to their promise as easy ways to promote health and avoid disease.
